# Understanding the Transcription Factor NFE2L1/NRF1 from the Perspective of Hallmarks of Cancer

**DOI:** 10.3390/antiox13070758

**Published:** 2024-06-22

**Authors:** Haomeng Zhang, Yong Liu, Ke Zhang, Zhixuan Hong, Zongfeng Liu, Zhe Liu, Guichen Li, Yuanyuan Xu, Jingbo Pi, Jingqi Fu, Yuanhong Xu

**Affiliations:** 1 Key Laboratory of Environmental Stress and Chronic Disease Control and Prevention, Ministry of Education, China Medical University, No. 77 Puhe Road, Shenyang North New Area, Shenyang 110122, China; 2 Department of Pancreatic and Biliary Surgery, The First Affiliated Hospital, China Medical University, No. 155 Nanjing North Street, Heping District, Shenyang 110001, China; 3 Program of Environmental Toxicology, School of Public Health, China Medical University, No. 77 Puhe Road, Shenyang North New Area, Shenyang 110122, China; 4 Department of Nutrition and Food Hygiene, School of Public Health, China Medical University, No. 77 Puhe Road, Shenyang North New Area, Shenyang 110122, China; 5 Laboratory of Chronic Disease and Environmental Genomics, School of Public Health, China Medical University, No. 77 Puhe Road, Shenyang North New Area, Shenyang 110122, China; 6 Key Laboratory of Liaoning Province on Toxic and Biological Effects of Arsenic, School of Public Health, China Medical University, No. 77 Puhe Road, Shenyang North New Area, Shenyang 110122, China

**Keywords:** NFE2L1, hallmarks of cancer, cell proliferation, proteasome inhibitors, redox homoeostasis

## Abstract

Cancer cells subvert multiple properties of normal cells, including escaping strict cell cycle regulation, gaining resistance to cell death, and remodeling the tumor microenvironment. The hallmarks of cancer have recently been updated and summarized. Nuclear factor erythroid 2-related factor 1 (NFE2L1, also named NRF1) belongs to the cap’n’collar (CNC) basic-region leucine zipper (bZIP) family. It acts as a transcription factor and is indispensable for maintaining both cellular homoeostasis and organ integrity during development and growth, as well as adaptive responses to pathophysiological stressors. In addition, NFE2L1 mediates the proteasome bounce-back effect in the clinical proteasome inhibitor therapy of neuroblastoma, multiple myeloma, and triple-negative breast cancer, which quickly induces proteasome inhibitor resistance. Recent studies have shown that NFE2L1 mediates cell proliferation and metabolic reprogramming in various cancer cell lines. We combined the framework provided by “hallmarks of cancer” with recent research on NFE2L1 to summarize the role and mechanism of NFE2L1 in cancer. These ongoing efforts aim to contribute to the development of potential novel cancer therapies that target the NFE2L1 pathway and its activity.

## 1. Introduction

Cancer is the leading cause of death from disease and a major threat to life expectancy. In 2020, there were about 19.3 million newly diagnosed cancer patients in the world, and half of these cases will eventually lead to death [1]. Despite the rapid progress in early diagnosis, therapeutic strategies, and mechanism studies, the burden of cancer is expected to reach 28.4 million new cancer cases globally by 2040 [1]. Cancer cells subvert multiple properties of normal cells; thus, they not only escape strict cell cycle regulation, but also gain resistance to cell death [2]. In order to adapt to the tumor microenvironment, which normally lacks oxygen and nutrients, cancer cells reprogram their metabolism process to survive and sustain proliferation under great pressure [2,3].

To maintain cellular homeostasis and physiological integrity, all organisms living in oxygenated environments have evolved effective cytoprotective strategies to combat a wide range of stressors (oxidants, exogenous substances, and nutrients), pathophysiological stimuli, and other biological factors [4,5,6,7]. Numerous studies have shown that cap’n’collar (CNC) basic-region leucine zipper (bZIP) family members, Nuclear factor erythroid 2-related factor 2 (NFE2L2, also named NRF2) and the homologue Nuclear factor erythroid 2-related factor 1 (NFE2L1, also named NRF1), are the central regulatory factors in maintaining the resistance to oxidative and proteotoxic stress, as well as metabolic homeostasis in the basal state [8,9]. NFE2L1 loss-of-function and gain-of-function studies have shown that a series of pathophysiological alterations and diseases occurred in genetically modified *NFE2L1* cells and tissues. In addition, *Nfe2l1*-specific knockout in mouse liver, pancreatic β cells, brain, white adipose tissue, and osteoblasts and osteoclasts cause non-alcoholic steatohepatitis (NASH) [10,11], hyperinsulinemia [12], neurodegenerative disease [13,14], inflammation in white adipose tissue [15], and skeletal retardation [16,17], respectively. Recently, it has been suggested that NFE2L1 plays significant roles in the thermogenesis function of brown adipose tissue [18] and liver cholesterol homeostasis [19]. The role of NFE2L1 in the regulation of proteostasis in various human diseases has also drawn attention [20,21,22].

Furthermore, NFE2L1 levels are altered in a variety of cancers and are associated with the malignancy of several cancers. NFE2L1 is upregulated in liver adenocarcinomas [23]. However, downregulation of NFE2L1 has been found in ovarian and prostate cancers [24,25]. In melanoma, NFE2L1 was found to decrease with disease progression [26]. Kari’s group pointed out that a low nuclear location and high cytoplasmic location of NFE2L1 was associated with poor overall survival in diffuse large B-cell lymphoma [27]. In ovarian cancer, higher NFE2L1 levels were associated with shorter progression-free survival, and NFE2L1 was positively correlated with CD^8+^ T cell, macrophage, and neutrophil infiltration [25].

Functional normal cells convert into malignant cancer cells by reprogramming several biochemical and molecular processes. These alterations are considered to be hallmarks of cancer, which form a theoretical framework that has proven enduring utility for rationalizing the vast complexity of cancers and the underlying mechanisms [2,28,29]. As a transcription factor, NFE2L1 undoubtedly plays an important role in various cancers. Therefore, this review will provide a summary of the role of NFE2L1 in cancer and the underlying molecular mechanisms from the perspective of hallmarks of cancer.

## 2. Structure and Function of Human NFE2L1

The adaptive regulatory response networks to stressors are primarily maintained by the CNC-bZIP transcription factor family, including p45 NFE2 (nuclear factor erythroid lineage 2) [30]; NFE2-associated factors NFE2L1 (including its long isoforms NFE2L1α/TCF11 and short isoforms NFE2L1β/LCR-F1) [31,32,33], NRF2 [34], and NFE2L3 [35]; and the transcriptional repressors Bach1 [36] and Bach2 [37]. The mRNA and protein analysis of NFE2L1 demonstrated that it is widely expressed at a constant level in all tissues and throughout the whole development period. NFE2L1 forms a heterodimer with small musculoaponeurotic fibrosarcoma oncogene (sMAF) proteins. The NFE2L1/sMAF dimers recognize a DNA sequence motif termed antioxidant response elements (AREs) and promote downstream gene transcripts [38,39,40,41]. AREs comprise a 5′-RTGACnnnGC-3′ (R = A or G) core sequence and are located in the enhancer and promoter regions of various genes [42,43]. Chromatin immunoprecipitation (ChIP)-seq data identified 5′-RTGACTCAGC-3′ as the consensus binding site of NFE2L1 [44]. NFE2L1 binding elements were found in the promoter regions of genes involved in many physiological and pathological processes [10,45,46,47,48,49,50]. It is now widely recognized that NFE2L1 is a multifunctional protein that plays a key role in a variety of cellular functions.

### 2.1. Basic Structure of NFE2L1

**Figure 1:**
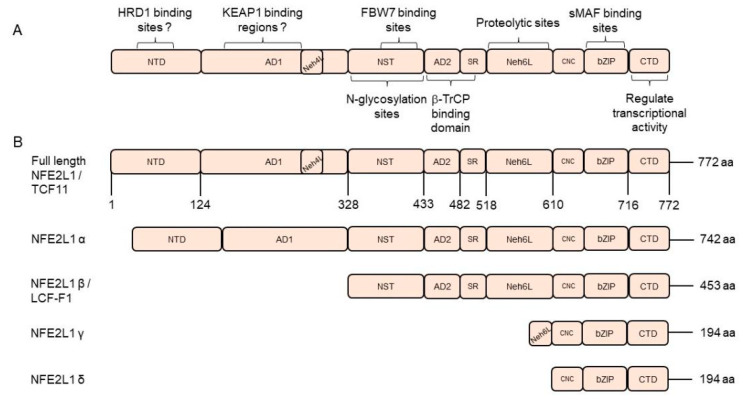
The structural domains and isoform of human nuclear factor erythroid 2-related factor 1 (NFE2L1). (**A**) Basic structural domains and correlated function of human NFE2L1. (**B**) Schematic protein structures of different isoforms of human NFE2L1. Abbreviations: NTD, N-terminal domain; AD1, acidic domain 1; NST, Asn/Ser/Thr-rich; AD2, acidic domain 2; SR, serine-repeat; Neh6L, Neh6-like; CNC, Cap ‘n’ collar; bZIP, basic-region zipper; CTD, C-terminal domain. All the sequences are from the National Center for Biotechnology (www.ncbi.nlm.nih.gov).

The human *NFE2L1* gene is located on chromosome 17q22 [32] and contains nine structural domains based on its sequence characteristics, namely, N-terminal domain (NTD), acidic domain 1 (AD1), asparagine/serine/threonine-rich (NST), acidic domain 2 (AD2), serine repeat (SR), Neh6L, CNC, bZIP, and C-terminal domain (CTD) [9,51]. N-terminal homology box 1 (NHB1) anchors NFE2L1 to the endoplasmic reticulum (ER), while NHB2 is a potential hydrolysis site of NFE2L1 that generates an N-terminal truncated protein form [52] (Figure 1A).

The human *NFE2L1* gene produces various protein isoforms through alternative splicing and selective use of translation initiation codons. Although their consensus sequences are similar, different isoforms may regulate the transcription of different genes, and even show combinatorial or competitive effect. There are three main isoforms of NFE2L1 gene in humans, including TCF11, NFE2L1α, and NFE2L1β/LCR-F1. The human full-length NFE2L1 isoform (TCF11) contains 772 amino acids (aa). However, TCF11 doesn’t exist in mice and only exists in humans, which has brought certain difficulties in conducting TCF11 function studies in mouse-related experiments. The human prototypic NFE2L1α (742 aa) lacks the Neh4L domain, due to alternative splicing of the TCF11 transcript to remove its exon 4 [53], and the two isoforms showed similar transcriptional activation activity [54,55]. NFE2L1α and TCF11 have similar yet different regulatory profiles, although both contribute basically to positive regulation of their co-targets [56]. In the process of translation, ribosomes can select different promoters to form LCR-F1, NFE2L1γ (36 kDa), and NFE2L1δ (25 kDa) [52,57]. LCR-F1 is a short isoform of NFE2L1, which contains 453 aa. LCR-F1 is also known as p65NFE2L1 due to its approximately 65 kDa in SDS-PAGE [58]. The ratio of the NFE2L1 isoforms is dependent on the cell types and altered in cancers. In normal hepatocytes, NFE2L1α is equivalent to TCF11, while NFE2L1α in liver cancer cells is about 90% [52]. In human erythroleukemia cells (K562), TCF11 and NFE2L1α are not detectable, but NFE2L1β exists [31]. NFE2L1β/LCR-F1, a significant negative inhibitor of ARE-driven gene transcriptional activation against wild-type NFE2L1 and NRF2 [59], lacks N-terminal AD1 and shows weak transcription activity [33,52,55]. In addition, the full-length NFE2L1 and its shorter isoform LCR-F1/NFE2L1β are inhibited by small dominant-negative NFE2L1γ/δ isoforms, which downregulate the expression of NF-E2/AP1-like ARE-driven genes [57,60] (Figure 1B).

### 2.2. Regulation of NFE2L1 Activation

**Figure 2:**
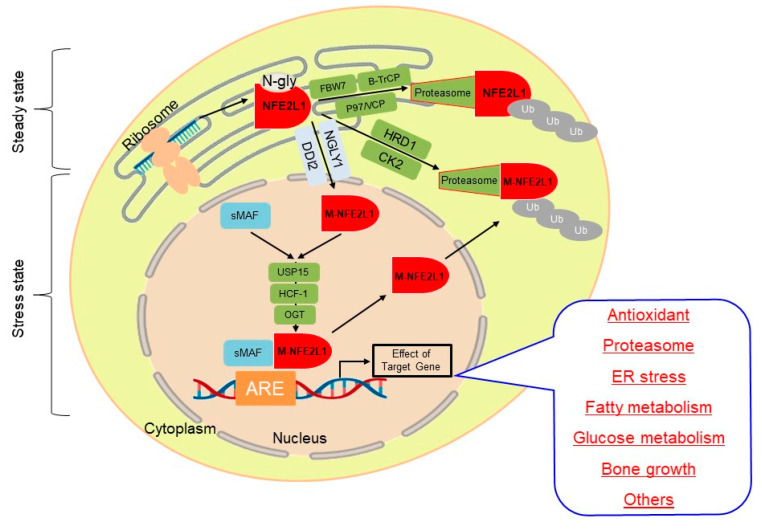
Proposed activation pathway of Long-Nuclear factor erythroid 2-related factor 1 (L-NFE2L1). NFE2L1 is inserted into the endoplasmic reticulum (ER), and its asparagine/Serine/Threonine-rich (NST) domain is N-glycosylated so as to become an inactive NFE2L1 glycoprotein. When required for induction by biological cues, the ER-protected transactivation domains of NFE2L1 are dynamically retrotranslocated via the AAA protein p97/valerian-containing casein (VCP) and repositioned from the luminal side of ER membranes into the cyto/nucleo-plasmic side, whereupon it undergoes various post-translational modifications, such as N-deglycosylation, O-GlcNAcylation, de-GlcNAcylation and phosphorylation to yield active isoforms. In this process, diverse enzymes like PNGase, O-Linked N-acetylglucosamine transferase (OGT), DNA damage inducible 1 homology 2 (DDI2), beta-transducin repeat-containing protein (β-TrCP), and ubiquitin ligase HMG-CoA reductase degradation 1 (HRD1) are involved. There are two distinct (β-TrCP- and HRD1-dependent) degradation mechanisms regulating NFE2L1 protein levels. In the cytoplasm, NFE2L1 is degraded and suppressed by the ER-associated degradation ubiquitin ligase HRD1 and VCP. NFE2L1 is also degraded in the nucleus via β-TrCP-mediated degradation. In cells with insufficient proteasome capacity, active NFE2L1 accumulates and then migrates to the nucleus, where it heterodimerizes with cofactors to bind ARE to induce gene transcription. In contrast, complete proteasomal processing of NFE2L1 may lead to decreased NFE2L1 protein levels and transcriptional activity.

NFE2L1 is regulated at multiple levels, including mRNA transcription and post-translational modification. NFE2L1 is initially an ER-localized protein, consisting of a C-terminus located in the ER lumen and a small N-terminal portion protruding into the cytoplasm [61,62,63]. As shown in Figure 2, in the ER lumen, NFE2L1 undergoes N-glycosylation [52,58,62,64]. Under steady-state conditions, NFE2L1 undergoes ER-associated degradation (ERAD), a pathway specifically designed to degrade misfolded ER proteins [65]. This process involves E3 ubiquitin ligase and homohexamer AAA-ATPase p97 (also known as valerian-containing casein, VCP) [63,66]. When the cells are exposed to ER stress, including proteasome inhibitors treatments, the C-terminal of NFE2L1 is translocated from ER lumen to the cytoplasm via p97/VCP and then de-glycosylated by NGLY1 [67,68]. After de-glycosylation, NFE2L1 is cleaved by DNA damage inducible 1 homolog 2 (DDI2), and the processed NFE2L1 and sMAF form heterodimers that bind to ARE sequences and promote specific downstream target gene expression [39,40,41,69]. During the ER-to-nuclear translocation of NFE2L1, a portion of its deglycoprotein is subject to ubiquitination by an ER membrane-bound E3 ligase HRD1 (also called synoviolin) and subsequent deubiquitination by ubiquitin-specific proteases (USPs) before being degraded by 26S proteasomes [70].

The human NFE2L1 protein is phosphorylated at the Ser350 and Ser448/451 sites, allowing it to interact with the Fbw7 and β-TrCP ubiquitin E3 ligase, respectively. After that, it is degraded by the proteasome [70,71]. NFE2L1 is also phosphorylated by casein kinase 2 at residue Ser497 to reduce the transcriptional activity [72]. The ubiquitin-specific protease 15 (USP15) enzyme has been shown to counteract Skp1-Cul1-F-box protein (SCF) with β-TrCP by deubiquitinating and stabilizing nuclear NFE2L1. In addition, nuclear NFE2L1 is also a substrate for O-GlcNAcylation glycosylation modification, and O-linked *N*-acetylglucosamine transferase (OGT) and host cell factor C1(HCF-1)-mediated NFE2L1 antagonize phosphorylation and inhibit NFE2L1 at the Ser448/451 site O-GlcNAcylation interaction with β-TrCP, leading to NFE2L1 accumulation in the nucleus and transactivation of its target genes [73]. The most well-understood aspect of post-modification is focused on the full length of NFE2L1. However, the short isoforms from mRNA translation remain undetermined.

### 2.3. The Downstream Target Genes of NFE2L1

NFE2L1 plays multiple roles in embryonic development, pancreatic β-cell function [74], osteoblast differentiation [16], maintenance of normal neuronal cell function [14], and adipogenesis and differentiation [75,76,77]. The detailed physiological function of NFE2L1 has been reviewed previously [9,78]. In brief, the global disruption of *Nfe2l1* leads to embryonic lethality due to anemia from impaired fetal liver erythropoiesis and severe oxidative stress at an early development stage in mice [45,46,79,80], indicating that this gene is indispensable for normal development, healthy growth, and defense from oxidative stress. Liver-specific *Nfe2l1* deficiency in adult mice leads to severe NAFLD, even hepatocellular carcinoma [10]. Hepatocytes *Nfe2l1* lacking in fetal mice show impaired expression of antioxidant genes and increased oxidative stress [81]. Regarding the specific mode of action, the transcription factor NFE2L1 is often compared with its homology NRF2 [82]. *Nrf2*-knockout mice do not develop spontaneous cancer or other phenotypic manifestations [83]. Moreover, *Nfe2l1* and *Nrf2* double-knockout embryo survival is less than either single knockout [46]. Mouse embryonic fibroblasts (MEFs) lacking both *Nfe2l1* and *Nrf2* result in increased intracellular ROS and cell death under normal oxygen conditions. However, low-oxygen (5% O_2_) culturing dramatically reduces cell death of double-knockout MEFs [46], suggesting functional redundancy and compensation between NFE2L1 and NRF2 in early mouse development and regulation of cellular ROS levels. Recently, Zhang’s group found that deletion of long-isoform *NFE2L1* caused increased oxidative damage, which cannot be eliminated by NRF2 activation and antioxidant enzyme enhancement [84]. This evidence proves that NFE2L1 has an important role in the regulation of redox homeostasis, as well as NRF2. Osteoblast *Nfe2l1* deficiency also causes osteoarthritis because of an imbalance in redox homeostasis, mainly due to reduced expression of antioxidant genes, including MT2, GCLC, and GCLM in osteoblast cells [85]. The downstream genes of NFE2L1 are involved in antioxidant defense [11,48,60,80,86,87,88,89,90], proteasome homeostasis [66,91], ER stress [92], fatty metabolism [89,93], glucose metabolism [12,74], and bone growth [94,95], which are summarized in Table 1. The above functions of NFE2L1 have been illuminated in genetically modified mouse models and cell lines. By using sequencing technology and bioinformatics methods, the new physiological function and novel downstream genes of NFE2L1 can be discovered [96,97].

### 2.4. NFE2L1 Maintains Redox Homeostasis in Cancer Cells

Oxidative stress is an imbalance between the production of reactive oxygen species (ROS) and the clearance of ROS from the body [98]. Along with the occurrence of cancer, imbalance of redox homeostasis exists [99]. Glutathione (GSH) is an important regulator of intracellular redox homeostasis. In 1990s, it was found that NFE2L1 maintains GSH synthesis and concentration [80]. Myhrstad et al. proved that TCF11, a long NFE2L1 isoform, binds sMAF proteins to recognize the ARE site in the promoter of heavy chain of γ-glutamylcysteine synthetase (γ-GCS (h)), and regulates GSH accumulation [88]. The following studies found that NFE2L1 promotes different downstream target genes, such as *GCLC*, *GCLM*, *MT1*, and *MT2*, to participate in the regulation of redox homeostasis [100]. Recently, Wufuer et al. found that a series of antioxidant and detoxification genes (encoding HO-1, NQO1, GCLC, GCLM, GSR, GPX1, MT1E, and MT2) were involved in ROS clearance after tert-butylhydroquinone (tBHQ) stimulation in HepG2 cells [101].

In HepG2 cells, *NFE2L1* deficiency leads to a significant increased ROS levels in the presence and absence of glucose, which indicates that partial inactivation of NFE2L1 aggravates the oxidative damage level [102]. Furthermore, the expression of antioxidant and detoxification genes was blunted in *NFE2L1* knockdown HepG2 cells [11,90,101,103]. Metformin, a first-line drug for type 2 diabetes therapy, induced ROS accumulation by inhibiting NFE2L1 expression in HepG2 cells [104]. Recently, by deletion of full-length *Nfe2l1* in the HepG2 cells, Zhang et al. found that glycolysis of *Nrf1α*^–∕–^ cells is enhanced to aggravate its mitochondrial pressure with increased ROS production. However, *Nrf1α*^–∕–^ leads to inactivation of the mitochondrial stress response, albeit NRF2 is hyperactive. In addition, the EMT-relevant signaling pathways are constitutively activated in *Nrf1α*^–∕–^ cells [84]. In prostate cancer, excessive ROS induce NFE2L1 activation and regulate downstream antioxidant genes, such as *peroxiredoxin 1* (*Prx-1*) and *thioredoxin 1* (*Txn-1*), which compensate for oxidative stress and maintain redox homeostasis [105]. More and more studies have confirmed that NFE2L1-mediated downstream regulatory genes play an important role in regulating redox homeostasis, as well as in cancer cells.

## 3. NFE2L1 and the Hallmarks of Cancer

“Hallmarks of Cancer: New Dimensions” was expanded by Professor Douglas Hanahan in 2022 to include up to 14 characteristics of cancer [29]. The 14 core hallmark capabilities are well established as having applicability across the spectrum of human cancers, and the cellular and molecular mechanisms by which the hallmarks are acquired are increasingly considered to be diverse. These cellular and molecular mechanisms are the biggest challenges facing cancer treatment. In addition, oncology research takes a similar approach.

**Figure 3:**
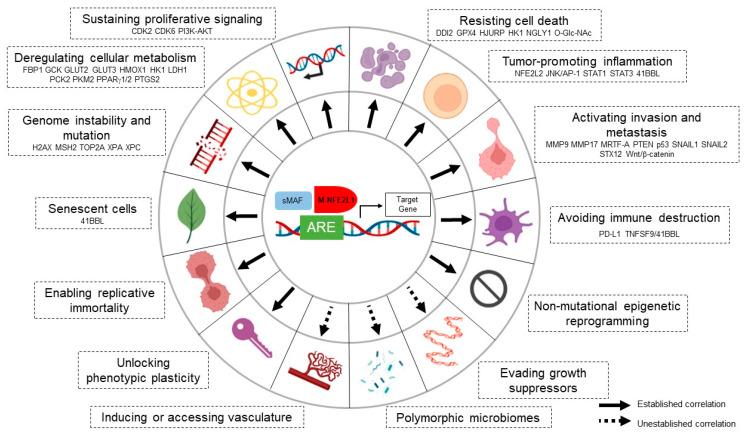
NFE2L1 regulated the hallmarks of cancer. NFE2L1 associates with cancer hallmarks traits through various biological features. The hallmarks with dashed arrow indicate the lack of scientific evidence. The illustration was regrated based on an updated review by Hanahan et al. [29].

Over the past decade, studies have found NFE2L1 is involved in the regulation of key processes related to the hallmarks of cancer, including tumor growth, invasion, migration and metastasis, cell death, genomic instability, reprogramming cell metabolism, inflammation, and cell senescence. Although other hallmarks have not been studied in cancers, enormous research potential has also been found in normal cells. Here, we have organized an interaction diagram between NFE2L1 and hallmarks to facilitate understanding of the research progress of NFE2L1 in cancers (Figure 3).

### 3.1. Sustained Proliferative Signaling

The most basic characteristic of cancer cells is their ability to sustain chronic proliferation. By deregulating normal tissues, cancer cells control the production and release of growth-promoting signals, causing uncontrolled cell growth. Zhang’s group made a series of attempts to interpret the role of NFE2L1 in cancer cell proliferation. In HepG2 cells, *NFE2L1* mRNA silencing using shRNA caused malignant growth with the shortened G1 phase of cell cycles [106,107]. Knockout *NFE2L1α* and *TCF11* resulted in the same phenotype as all deficient *NFE2L1* isoforms. Knockout of *NFE2L1α* caused downregulation of the p53-p21-CDK2 signaling pathway and upregulation of CDK6, Cyclin D1, and p16 cyclin-dependent kinase inhibitor 2A, which arrest the cell cycle at G2/M phases and inhibit apoptosis [108]. Both phosphorylated proteins of PI3K (p110) and AKT were also significantly upregulated and contributed to uncontrolled cell proliferation in *NFE2L1α*-knockout cells [84]. Loss of *NFE2L1α* leads to hyperactivation of NRF2 [109]. In a xenograft mouse study, malignant growth of *NFE2L1α*-knockout HepG2 cells was almost eliminated by *NRF2* silencing. However, the tumor promotion by hyperactivation was efficiently confined by NFE2L1α, which serves as a dominant tumor repressor [109]. TCF11 and NFE2L1α exhibited a similar regulatory profile, but with some isoform-specific target genes. These specific targets maintain TCF11 as a more potent tumor repressor than NFE2L1α, although they both prevent tumor development and malignant growth in HepG2 cells [56]. Furthermore, in liver-specific knockout mice, *Nfe2l1* depletion also resulted in hepatoma formation, including hepatocellular carcinoma and adenoma [10].

However, current research on cancer proliferation is mainly conducted in liver cancer, and the role of NFE2L1 in other types of cancers is not yet clear. Whether NFE2L1 inhibits cancers in all cancers or whether the role played by NFE2L1 depends on the type and stage of the cancer, as in NRF2, is still uncertain.

### 3.2. Resistance to Cell Death

Cancer cells have an excessive production of protein due to epigenetic alterations, gene fusions, or amplifications and increased metabolic rates [110]. In addition, genomic instability and altered redox states lead to the abnormal production of proteins and protein misfolding, which enhance the proteotoxic stress in cancer cells [110]. Eukaryotic systems have developed intracellular protein quality control (PQC) mechanisms [107,108,109]. In the PQC maintenance, the ubiquitin (Ub)-proteasome system (UPS) is a critical proteolytic pathway for selectively eliminating Ub-tagged substrate proteins in eukaryotes [109]. Since several tumors, including hematological malignancies and lung, breast, pancreatic, head and neck, and thyroid cancers, are dependent on increased proteasome activity, the proteasome inhibitors are considered as effective tumor therapy, which can induce proteotoxic stress-mediated cancer cell death [111,112,113,114,115]. Proteasome inhibitor therapy have been widely used in multiple myeloma (MM) and other hematological diseases [116], which is approved by the US Food and Drug Administration [117].

Proteasome inhibition leads to the disturbance of NF-κB signal transduction, stabilization of p53, cell cycle arrest, and accumulation of oxidized proteins in cancer cells, all of which lead to apoptosis [118]. When proteasome activity is reduced or inhibited by proteasome inhibitors, proteasome subunit gene transcription is activated, which lead a rapid acquisition of resistance to proteasome inhibitors in most cancers [119]. This phenomenon is known as the “bounce-back” effect, which is mediated by NFE2L1 nuclear translocation, accumulation, glycosylation, and downstream proteasome subunit gene expression [66,73,91]. Therefore, the strategy of NFE2L1 activity inhibition has been used to sensitize the resistance of proteasome inhibitor treatments in cancers.

In a MM cell line study, bromodomain extra-terminal (BET) protein inhibitors were synergized with proteasome inhibitors (carfilzomib, CFZ) to accelerate proteotoxic stress-mediated cell death in vitro, by inhibiting the NFE2L1-mediated “bounce-back” effect [120]. In proteasome inhibitor therapy of MM, *DDI2* knockout blocks NFE2L1 cleavage and nuclear translocation, which increases the sensitivity of proteasome inhibitor (CFZ)-induced cytotoxicity both in vitro and in vivo [121]. In MEF cells, inhibition of NGLY1 enhanced the cytotoxicity of proteasome inhibitors (CFZ), through greatly reducing NFE2L1 entry into the nucleus [67]. Furthermore, a similar effect is also observed in solid tumor cell lines. In TNBC cell line MDA-MB-231, the protease *DDI2* deficiency attenuates NFE2L1 protein cleavage and activation, then enhances the cytotoxicity induced by proteasome inhibitor drugs (CFZ) [122].

The combination therapy of PIM kinase inhibitor and 20S proteasome inhibitor was utilized in triple-negative breast cancer (TNBC) cell lines. PIM kinase inhibitor accelerated NFE2L1 degradation, and then avoided the “bounce-back” effect after 20S proteasome inhibitor treatment [123]. In another TNBC study, proteasome subunit cleavage site-specific inhibitor treatments and CRISPR/Cas9-mediated genetic inhibition of proteasome subunit cleavage site-suppressed NFE2L1 activity and mediated proteasome subunit induction, and prevented “bounce-back” effect in vitro and in vivo [124]. Six-O-carboxypropyl-α-tocotrienol (α-T3E), a redox-silent analogue of tocotrienols, was shown to disrupt proteasome homeostasis by inactivating NFE2L1, eventually inducing severe ER stress and increasing the sensitivity of chemoresistance (cisplatin) in malignant mesothelioma cells [125].

In myeloma or neuronal cell lines, low concentrations of proteasome inhibitors (Bortezomib, BTZ, 10 nM) induced cell death by inhibiting proteasome activity. However, high concentrations of proteasome inhibitors (BTZ, 10 μM) prevented NFE2L1 degradation in the ER, and then promoted the 26S proteasome subunit family expression to compensate for the proteasome activity inhibition [126]. During radiation therapy of hypopharyngeal carcinoma, transcription factor NFE2L1-mediated proteasome gene expression was increased, promoting degradation of DEP domain-containing mTOR-interacting protein (DEPTOR) and resulting in mTORC1 signaling activation and radiotherapy resistance. Proteasome inhibitor (BTZ) combination therapy was used to sensitize hypopharyngeal carcinoma to radiotherapy [127].

Besides proteasome inhibitor cancer therapy, NFE2L1 also regulates other types of chemotherapy-induced regulated cell death (RCD), including apoptosis, ferroptosis, and genotoxic stress-induced cell death. Pancreatic β cell *Nfe2l1*-knockout mice showed tolerance to STZ-induced apoptosis [128]. In a transplantation tumor model, *Nfe2l1*-KD insulinoma MIN6 cells grew faster and developed resistance to streptozotocin (STZ) and 5-fluorouracil. Overexpressed HK1 in *Nfe2l1*-KD insulinoma cells attached to the mitochondria to block mitochondrial toxicant-induced cytotoxicity and apoptosis [129].

It has been progressively recognized that several oncogenic pathways are closely associated with ferroptosis [130]. In the human lung cancer cell A549, the NGLY1/NFE2L1 pathway promotes GPX4 protein expression to inhibit ferroptosis. Disruption of NGLY1 or NFE2L1 reduces GPX4 protein expression, and overexpression of GPX4 rescues ferroptosis susceptibility in NFE2L1 mutants [131]. NFE2L1 restrains ferroptosis by transcriptionally regulating the Holliday junction recognition protein (HJURP) and participates in the progress of Oral Squamous Cell Carcinoma (OSCC) [132]. In addition, NFE2L1 prevents ferroptosis by maintaining proteasomal activity [133].

In summary, NFE2L1 in cancer cells plays an important role in resisting cell death, including apoptosis, pyroptosis, and ferroptosis. With more research, the role of NFE2L1 in other emerging RCDs will be discovered. Given the extensive clinical research on drugs targeting RCD, the application prospects of NFE2L1 in this field are broad.

### 3.3. Metabolic Reprogramming

NFE2L1 is crucial for maintaining homeostasis of glucose, lipids, and protein metabolism. In physiological conditions, both NFE2L1 overexpression and pancreatic β cell-specific deletion mouse models showed impaired glucose metabolism [12,74]. Liver-specific *Nfe2l1* knockout leads to steatohepatitis spontaneously, via regulating several lipid metabolism genes, namely, *Lipin1*, *Pgc-1*, *Apoer2*, *Vldlr*, *Fads3*, and *Alox5ap* [10,89,93]. During high-cholesterol diet feeding, NFE2L1 mediated cholesterol output disorder and triggered inflammatory signal transduction by regulating *CD36* and *LXR* [134,135]. Metabolite sensing and signaling in mTORC1-SREBP1 promotes *Nfe2l1* expression, contributing to AMPK kinase activity and the following protein synthesis and turnover in the liver [136,137,138].

In cancers, glucose metabolic reprogramming was firstly documented as the Warburg effect by Otto Warburg in 1920s. The Warburg effect is characterized as enhanced glycolysis rate. Oxidative phosphorylation in cancer cells decreases, while aerobic glycolysis pathway increases, independent of the presence of oxygen [139]. Enhanced catabolism of glucose contributes to synthesis of nucleotides, amino acids, and lipids from the raw metabolites to support energy and uncontrolled tumor growth [140,141]. The following studies found that metabolic reprogramming exists in a variety of cancer cells, involving the anabolism and catabolism of carbohydrates, as well as proteins and lipids [2]. Typically, tumor cells usually increase glucose and glutamine uptake. Therefore, this metabolic rate increase constitutes one of the typical characteristics of tumor cells [2]. The survival of cancer cells also depends on cellular metabolism. Recent studies suggest that a low glycolysis rate leads to increased apoptosis [142], and increased glycolysis effectively inhibits apoptosis [143,144].

Accumulating studies demonstrate that NFE2L1 is also crucial for metabolic reprogramming in various cancers. In HepG2 cells, *NFE2L1* deficiency leads to the typical Warburg effect and exacerbates glucose metabolism and ATP production by increasing glucose uptake the and glycolytic pathway [102]. It was found that the mRNA levels of glucose transporter families and the glycolytic enzymes were increased, but the genes in charge of metabolic flow from glycolysis to the TCA cycle were decreased in *NFE2L1*-deficient HepG2 cells [12,74,102,145]. This indicated that *NFE2L1* deficiency enhanced glycolysis, impaired mitochondrial function, and inhibited oxidative phosphorylation [102]. In addition, several amino acids, including glycerate, valine, isoleucine, leucine, cysteate, cysteine, serine, glycine and threonine, were also significantly increased in *NFE2L1*-deficient HepG2 cells [102]. Besides glucose and amino acid metabolism, NFE2L1 also regulates lipid metabolism in cancer. The pyruvate kinase M2 isoform (PKM2) regulates anabolic metabolism and lipid homeostasis in cancer cells. In breast cancer cells, PKM2 controls NFE2L1 cleavage and the transcriptional downstream transmembrane protein 33 (TMEM33), which regulates activation of SREBPs and lipid metabolism [146]. The further study proved that *NFE2L1* deficiency leads to activation of the PI3K-AKT-mTOR signaling pathway, which may contribute to the increased expression of critical genes of the glucose uptake, glycolysis, the pentose phosphate pathway, and the de novo lipid synthesis in HepG2 cells [147].

Overall, the uncontrolled growth of tumor cells requires higher-energy metabolism to maintain. NFE2L1 plays a huge role in the metabolic reprogramming of cancers. As an intracellular energy receptor, NFE2L1 extensively regulates the three major nutrients in cells, including glucose metabolism, lipid metabolism, and amino acid metabolism. It can be considered that the disturbance of NFE2L1 is related to the survival and death of cancer cells.

### 3.4. Tissue Invasion and Metastasis

In human hepatocellular carcinoma (HepG2) cells, NFE2L1α inhibits cancer cell migration and invasion [84,108]. Wnt/β-catenin-dependent and -independent signaling networks were significantly inhibited in *NFE2L1*-deficient hepatocellular carcinoma HepG2 cells [106]. It has also been shown that *NFE2L1* deficiency caused inactivation of two tumor repressors PTEN and p53, concurrently with oncogenic activation PI3K-PDK1-AKT signaling [106]. *NFE2L1α*-knockout HepG2 cells resulted in EMT progression, via impairing expression of two key mediators of EMT (Snail1 and Snail2) [108]. Moreover, loss of *NFE2L1α* also upregulated transcriptional expression of genes encoding matrix metallopeptidase 9 (MMP9) and membrane-type MMP17, which promotes cell invasion and migration [108]. Lee et al. showed that mitochondrial respiratory defects mediated NFE2L1 upregulation, which promotes the expression of its effective downstream target syntaxin 12 (STX12), a key regulator of cancer progression in several liver cancer cell lines [23].

In breast cancer MDA-MB-231 cells, Xu et al. found that NFE2L1α inhibited cancer cell migration and invasion. Furthermore, both NFE2L1α and MRTF-A are in a novel regulation loop, in which NFE2L1α inhibits the expression of MRTF-A via miR-219, and MRTF-A binds to the CarG box in the promoter of *NFE2L1α* and promotes its expression [148]. However, long-isoform NFE2L1 promotes cancer invasion and metastasis under chronic stress conditions. In human bronchial epithelial BEAS-2B cells, long-term arsenite exposure induced long-isoform NFE2L1 upregulation, which promotes EMT by positively regulating Snail1 transcription [149]. Gene set enrichment analysis and prognosis analysis indicated that NFE2L1 could be a key transcription factor in the acquisition of metastasis potential in ovarian cancer cells [150].

Based on the summary in this section, the role of NFE2L1 in invasion and metastasis is not identical in different types of tumors, and further research is needed to explore this hallmark.

### 3.5. Tumor-Promoting Inflammation

The tumor microenvironment is a complicated environment, including tumor cells and surrounding immune cells, fibroblasts, signaling molecules, and the extracellular matrix (ECM). It influences tumor progression and prognosis, as well as the effectiveness of cancer therapies. Several studies have shown that NFE2L1 plays the role of inhibiting inflammation response only in cancer cell line and immune cell line models. However, the role of NFE2L1 in the microenvironment of tumor tissues is not undetermined.

In *NFE2L1α*-knockout HepG2 cells, the inflammation marker COX2 was upregulated by increasing NRF2 and JNK/AP-1 pathways [109]. A series of cytokine mRNA expression, encoding IL-1A, IL-1B, IL-1R1, IL-1R2, IL-6, IL-8, IL-10, TGF1α, and TGF1β, was also significantly elevated [109]. Wang et al. found that *Nfe2l1* long isoform deficiency in macrophages led to M1 polarization via STAT1 and STAT3 activation, but not NF-κB, ERK1/2, or P38 signaling pathways [49]. It was also shown that TCF11/MAFG bind at the iNOS promoter to repress the induction of iNOS gene expression by cytokines [151]. Both NFE2L1 and other AP1 components interact with the promoter of TNFα and stimulate its transcription in mouse mast cells and dendritic cells [60,152].

### 3.6. Evading Immune Destruction

Immunosuppression is an important reason of cancer cell proliferation and growth, and the recovery of the immune system is an important strategy for cancer therapy [153]. The 41BBL protein is a possible oxidative stress-responsive immune checkpoint, which was directly regulated by NFE2L1 in HepG2 human liver cancer cells [107]. Furthermore, in breast cancer cells, it was demonstrated that NFE2L1 played the critical role in evading T-cell killing by upregulating super-enhancer-mediated PD-L1 expression [154].

### 3.7. Genome Instability

*NFE2L1* deficiency leads to the instability of the genome and malignant proliferation and growth [155]. An increase in micronuclei formation and chromosomal instability is observed in the *Nfe2l1*-deficiency mouse model [10]. *NFE2L1* deficiency in SAOS-2 cells resulted in increased micronuclei, abnormal mitosis, and multinucleated cells by decreasing the expression of various genes encoding kinetochore and mitotic checkpoint proteins [156]. In addition, NFE2L1 has also been reported to play an important role in DNA repair in response to UVB irradiation by regulating the expression of xeroderma pigmentosum C (XPC) in HaCat cells [155], as well as in CDDP-induced DNA damage and repair cascades by regulating *H2AX*, *XPA*, *TOP2A*, and *MSH2*, in HepG2 cells [157].

### 3.8. Cell Senescence

Senescent cells have been defined as one of the hallmarks of cancer [29]. Cellular senescence is a typically irreversible form of proliferative arrest, ostensibly as a complementary mechanism to programmed cell death that serves to inactivate and, in due course, remove diseased, dysfunctional, or otherwise unnecessary cells. The differentially expressed gene enrichment results indicated that senescence is the primary way to inhibit cell proliferation in the HepG2 cells to overexpress TNF superfamily member 9 (TNFSF9, also known as 41BBL or CD137 L) regulated by NFE2L1 [107]. Recently, a study demonstrated a new therapeutic pathway for cellular senescence protection against oxidative damage by delivering circLRRC8A-enriched exosomes through the circLRRC8A/miR-125a-3p/NFE2L1 axis for therapeutic premature ovarian failure (POF) [158]. Establishing a method to accelerate senescent cells can provide a new direction for cancer treatment. NFE2L1 surveys cellular protein folding and adjusts proteasome capacity to meet the demands of protein quality control pathways. This regulatory axis is critical for healthy aging [159].

### 3.9. Unlocking Phenotypic Plasticity

Cellular plasticity is not a novel invention or aberration of cancer cells, but rather the corruption of latent but activatable capabilities that various normal cells use to support homeostasis, repair, and regeneration [160]. Dedifferentiation of mature cells to their progenitor state, developmental cells that arrest differentiation to preserve a progenitor/stem cell state, and trans-differentiation into alternative cell lineages all appear to play a role in various types of cancer during primary tumor formation, malignant progression, and/or treatment response [29]. Currently, NFE2L1 regulates cell differentiation in non-tumor cells. A novel reported nonsense variant of NFE2L1 with diminished activity results in developmental delay, hypotonia, genital abnormalities, and growth retardation in patients [161]. NFE2L1 promotes heart regeneration and repair by regulating proteostasis and redox balance [162]. Nfe2l1 deficiency disturbed the expression of lipolytic genes in adipocytes, leading to adipocyte hypertrophy followed by inflammation, pyroptosis, and insulin resistance [77]. NFE2L1 regulates osteoclast differentiation in antioxidant-dependent and independent manners [17]. NFE2L1 plays an important role in tissue differentiation, and differentiation is closely related to unlocking phenotypic plasticity in the hallmarks.

## 4. Conclusions

**Figure 4:**
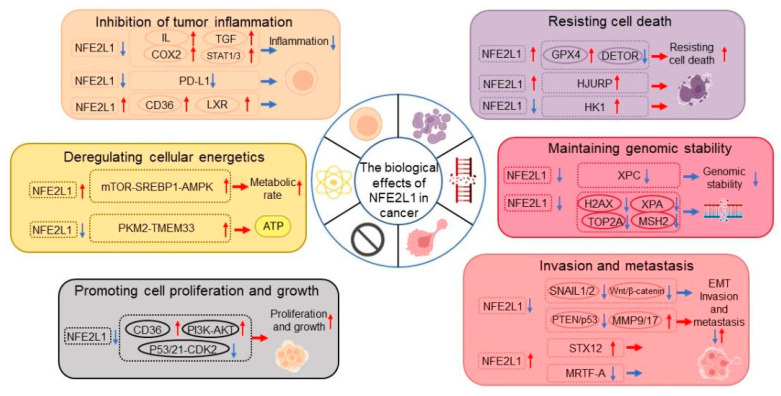
NFE2L1 regulates cancer cell malignance. In this review, we mainly focus on six hallmarks of cancer regulated by NFE2L1, including resisting cell death, enabling replicative immortality, activity invasion and metastasis, proteotoxic stress, deregulating cellular energetics and anti-oxidant/altered redox balance, as well as the underlying mechanisms.

It is not surprising that NFE2L1 studies continue to grow each year, since new modes of NFE2L1 regulation and new functions (i.e., target genes) are constantly being described. This review presents evidence of the tumor-suppressing and tumor-promoting effects of NFE2L1 according to its roles in nine hallmarks of cancer (Figure 4). The remaining hallmarks, although not yet reported in cancers, have shown huge research potential in their role in normal tissues.

In terms of cells and mouse models, the mystery of NFE2L1 has been revealed step by step [78]. The indispensable biological traits of NFE2L1 have been established and accepted. However, the appeal of NFE2L1-related cellular and molecular mechanisms consists in different isoforms and SNPs, which are still under investigation. Recently, NFE2L1-related isoforms and SNPs serve as potential therapeutical targets for neurodegenerative diseases [163] and cancer [164], and are receiving increased attention. The value of NFE2L1 for developing precise cancer treatment is immeasurable.

## Figures and Tables

**Table 1:** Downstream or correlation target gene of nuclear factor erythroid 2-related factor 1(NFE2L1) regulation.

Cellular Functions	Downstream or Correlation Target Gene	Up/Down	Notes
Antioxidant defense	*GCLC* [88], *GCLM* [80], *GST* [48], *HO-1* [90], *NQO1* [60]	Up	Recognition of ARE site
	*xCT* [89]	Down	
	*MT1/2* [11]	Down	Deletion of *NFE2L1*
Proteasome homeostasis	*PSMA3*, *PSMB6*, *PSMC4*, *PSMD1*, *PSMD12* [66,91]	Up	Recognition of ARE site
Fatty metabolism	*Apoer2*, *Vldlr*, *Fads3*, *Alox5ap* [89]	Up	Recognition of ARE site
	*Ldlr* [89]	-	
	*Lipin1*, *PGC-1β* [93]	Down	
ER stress	*ATF4*, *Gadd45b*, *CHOP* [92]	Up	Deletion of *NFE2L1*
Glucose metabolism	*HK1* [12]	Up	Deletion of *NFE2L1*
	*Slc2a2*, *Gck* [74]	Down	Inhibition of *NFE2L1*
Bone growth	*Osterix* [94]	Up	Recognition of ARE site
	*DSPP* [95]	Down	

## Abbreviations

AD1	acidic domain 1
AD2	acidic domain 2
AP1	activator protein 1
ARE	antioxidant response element
ATF3	activating transcription factor-3
Bzip	basic region leucine zipper
BET	bromodomain and extra-terminal
BTZ	bortezomib
CFZ	carfilzomib
CNC	cap ‘n’ collar
CZM	capzimin
CK2	casein kinase 2
CtBP2	c-terminal binding protein 2
CTD	c-terminal domain
CRLs	cullin-RING ubiquitin ligases
DEPTOR	degradation of mTOR-interacting protein
ECM	extracellular matrix
EMT	epithelial-mesenchymal transition
ER	endoplasmic reticulum
ERAD	er-associated degradation
GCLC	glutamate-cysteine ligase catalytic
GCLM	glutamate-cysteine ligase modifier
GCS	glutamyl cysteine synthetase
GSH	glutathione
GSR	glutathione-S-reductase
HO-1	heme oxygenase-1
HCC	hepatocellular carcinoma cancer
HepG2	hepatocellular carcinoma
HC	hypopharyngeal carcinoma
HCF-1	host cell factor C1
HIF-1	hypoxia-inducible factor 1
MEF	mouse embryonic fibroblast
MT	metallothionein
MM	multiple myeloma
MMP9	matrix metallopeptidase 9
NAFLD	nonalcoholic fatty liver disease
NASH	non-alcoholic steatohepatitis
NQO1	NAD(P)H: quinone oxidoreductase 1
NFκB	nuclear factor-kappaB
NFE2L1	nuclear factor erythroid 2-like 1
NFE2L2	nuclear factor erythroid 2-like 2
NHB1	n-terminal homology box 1
NST	asparagine/Serine/Threonine-rich
NTD	n-terminal domain
OGT	O-linked N-acetylglucosamine transferase
PPP	pentose phosphate pathway
PKM2	pyruvate kinase M2
POF	premature ovarian failure
PQC	protein quality control
Prx-1	peroxiredoxin 1
PSM	proteasome subunit family
mTORC1	rapamycin complex 1
ROS	reactive oxygen species
SCAP	SREBP-cleavage activating protein
SCF	skp1-Cul1-F-box protein
sMAF	small musculoaponeurotic fibrosarcoma
SNPs	single nucleotide polymorphism
SR	serine-repeat
SREBP1	sterol regulatory element binding protein 1
SSP	serine synthesis pathway
STX12	syntaxin 12
STZ	streptozotocin
tBHQ	tertbutyl hydroquinone
TCA cycle	tricarboxylic acid cycle
TMEM33	transmembrane protein 33
TNBC	triple-negative breast cancer
TNFSF9	TNF superfamily member 9
Txn-1	thioredoxin 1
USP15	ubiquitin-specific protease 15
UV	ultraviolet
UPR	unfolded protein response
UPS	ubiquitin-proteasome system
VCP	valerian-containing casein protein
XPC	xeroderma pigmentosum C
γ-GCS	γ-glutamylcysteine synthetase
